# Supplementary material for: Early Intervention Including an Active Motor Component in Preterms with Varying Risks for Neuromotor Delay: A Systematic Review and Narrative Synthesis
Source: J Clin Med. 2025 Feb 18;14(4):1364. doi: 10.3390/jcm14041364 (PMC11855987; doi:10.3390/jcm14041364)
Supplement: Supplementary file 1 [file jcm-14-01364-s001.zip › Supplementary material S1.pdf]

Supplementary material S1: search strategy

PUBMED

Search: (((Infant-premature OR infant-low birth weight OR infant- high risk Cerebral Palsy) AND (early intervention (education) OR developmental care OR physical therapy OR occupational therapy OR rehabilitation OR exercise OR neurodevelopmental therapy OR infant stimulation)) AND (child development OR infant development OR developmental disabilities OR psychomotor performance OR psychomotor disorders OR cerebral palsy OR developmental co-ordination disorder OR movement disorders OR motor skill disorders)) NOT (drug therapy OR genetics OR chest physiotherapy OR cardiac OR nutrition) **Filters:** Clinical Trial, Randomized Controlled Trial, from 2015/8/1, Sort by: Publication Date

WEB of SCIENCE

((#2 AND #1 AND #3) NOT #4) AND(TS=(randomised OR randomized OR randomisation OR randomisation OR placebo* OR (random* AND (allocat* OR assign*)) OR (blind* AND (single OR double OR treble OR triple))))

Filter: articles from 2015

Query #1
Infant-premature OR infant-low birth weight OR infant- high risk cerebral palsy (All Fields)
Edit
Query #2
early intervention (education) OR developmental care OR physical therapy OR occupational therapy OR rehabilitation OR exercise OR neurodevelopmental therapy OR infant stimulation (All Fields)
Edit
Query #3
child development OR infant development OR developmental disabilities OR psychomotor performance OR psychomotor disorders OR cerebral palsy OR developmental co-ordination disorder OR movement disorders OR motor skill disorders. (All Fields)
Edit
Query #4
drug therapy OR genetics OR chest physiotherapy OR cardiac OR nutrition (All Fields)

EMBASE

(prematurity/exp OR 'birth premature' OR 'infant, premature' OR 'infant, premature, diseases' OR 'neonate, premature' OR 'pre-mature birth' OR 'pre-mature infant' OR 'pre-maturity' OR 'pre-term babies' OR 'pre-term baby' OR 'pre-term birth' OR 'pre-term child' OR 'pre-term infant' OR 'pre-term infants' OR 'pre-term neonate' OR 'pre-term neonates' OR 'pre-term

newborn' OR 'pre-term newborns' OR 'premature' OR 'premature babies' OR 'premature baby' OR 'premature birth' OR 'premature child' OR 'premature childbirth' OR 'premature infant' OR 'premature infant disease' OR 'premature infant diseases' OR 'premature infants' OR 'premature neonate' OR 'premature neonates' OR 'premature newborn' OR 'premature newborns' OR 'premature syndrome' OR 'prematuritas' OR 'prematurities' OR 'prematurity' OR 'preterm babies' OR 'preterm baby' OR 'preterm birth' OR 'preterm child' OR 'preterm infant' OR 'preterm infants' OR 'preterm neonate' OR 'preterm neonates' OR 'preterm newborn' OR 'preterm newborns') OR ('low birth weight'/exp OR 'LBW baby' OR 'LBW infant' OR 'LBW neonate' OR 'LBW newborn' OR 'birth weight, low' OR 'infant, low birth weight' OR 'low birth weight' OR 'low birth weight infant' OR 'low birthweight' OR 'neonatal underweight') OR ('cerebral palsy'/exp OR 'brain palsy' OR 'brain paralysis' OR 'central palsy' OR 'central paralysis' OR 'cerebral palsy' OR 'cerebral paralysis' OR 'cerebral paresis' OR 'diplegia spastica' OR 'encephalopathia infantilis' OR 'palsy, cerebral' OR 'spastic diplegia') AND ('early childhood intervention'/exp OR 'head start program' OR 'early childhood intervention' OR 'early education intervention' OR 'early educational intervention' OR 'early intervention (education)' OR 'early intervention, educational' OR 'education early intervention' OR 'educational early intervention' OR 'developmental care'/exp OR 'occupational therapy' OR 'rehabilitation'/exp OR 'functional readaptation' OR 'medical rehabilitation' OR 'readaption' OR 'readjustment' OR 'rehabilitation' OR 'rehabilitation concept' OR 'rehabilitation engineering' OR 'rehabilitation potential' OR 'rehabilitation process' OR 'rehabilitation program' OR 'rehabilitation programme' OR 'rehabilitation, medical' OR 'rehabilitative treatment' OR 'resocialisation' OR 'resocialisation therapy' OR 'resocialization' OR 'resocialization therapy' OR 'revalidation' OR 'exercise'/exp OR 'biometric exercise' OR 'effort' OR 'exercise' OR 'exercise capacity' OR 'exercise performance' OR 'exercise training' OR 'exertion' OR 'fitness training' OR 'fitness workout' OR 'physical conditioning, human' OR 'physical effort' OR 'physical exercise' OR 'physical exertion' OR 'physical work-out' OR 'physical workout' OR 'neurodevelopmental therapy'/exp OR 'infant stimulation' OR 'cerebral palsy therapy'/exp) NOT ('drug therapy'/exp OR 'drug therapy' OR 'drug treatment' OR 'medicament therapy' OR 'medicament treatment' OR 'medication' OR 'medicinal therapy' OR 'medicinal treatment' OR 'pharmaceutical therapy' OR 'pharmaceutical treatment' OR 'pharmaco-therapy' OR 'pharmaco-treatment' OR 'pharmacological therapy' OR 'pharmacological treatment' OR 'pharmacotherapy' OR 'pharmacotreatment' OR 'therapeutic uses' OR 'therapy, drug' OR 'therapy, pharmacological' OR 'treatment, drug' OR 'treatment, pharmacological') NOT ('genetics'/exp OR 'genetic care' OR 'genetic research' OR 'genetics') NOT ('breathing exercise'/exp OR 'breathing exercise' OR 'breathing exercises' OR 'breathing therapy' OR 'chest physical therapy' OR 'chest physiotherapy' OR 'exercise, breathing' OR 'exercise, respiratory' OR 'respiration exercise' OR 'respiration therapy' OR 'respiratory exercise' OR 'respiratory physiotherapy') NOT 'cardiac'/exp NOT ('nutrition'/exp OR 'diet, food, and nutrition' OR 'nutrition' OR 'nutrition council' OR 'nutrition phenomena' OR 'nutrition physiology' OR 'nutrition processes' OR 'nutrition research' OR 'nutrition research center' OR 'nutrition study' OR 'nutrition survey' OR 'nutrition surveys' OR 'nutritional physiological phenomena' OR 'nutritional physiology' OR 'nutritive solution' OR 'sports

nutritional physiological phenomena') AND ('child development'/exp OR 'child development' OR 'development, child' OR 'infant development' OR 'developmental disability'/exp OR 'developmental disorder'/exp OR 'abnormal development' OR 'child development disorder' OR 'development disorder' OR 'developmental disabilities' OR 'developmental disorder' OR 'psychomotor performance'/exp OR 'psychomotor performance' OR 'psychomotor skill' OR 'psychomotor task' OR 'psychomotor disorder'/exp OR 'disturbance, psychomotor' OR 'motor coordination disorder' OR 'motor coordination dysfunction' OR 'motor skill disorder' OR 'motor skill dysfunction' OR 'motor skills disorder' OR 'motor skills disorders' OR 'motor skills dysfunction' OR 'psychomotor deficiency' OR 'psychomotor disorder' OR 'psychomotor disorders' OR 'psychomotor disturbance' OR 'psychomotor dysfunction' OR 'psychomotor impairment' OR 'psychomotoric disorder' OR 'cerebral palsy'/exp OR 'brain palsy' OR 'brain paralysis' OR 'central palsy' OR 'central paralysis' OR 'cerebral palsy' OR 'cerebral paralysis' OR 'cerebral paresis' OR 'diplegia spastica' OR 'encephalopathia infantilis' OR 'palsy, cerebral' OR 'spastic diplegia' OR 'developmental coordination disorder'/exp OR 'clumsy child syndrome' OR 'clumsy children' OR 'coordination disorder, developmental' OR 'developmental apraxia' OR 'developmental coordination disorder' OR 'developmental dyspraxia' OR 'developmental motor coordination disorder' OR 'developmental motor skill disorder' OR 'motor dysfunction'/exp OR 'disability, motor' OR 'motor disability' OR 'motor disfunction' OR 'motor disorder' OR 'motor disorders' OR 'motor disturbance' OR 'motor dysfunction' OR 'motor impairment' OR 'movement disorder' OR 'movement disorders') AND [2015-2023]/py AND [infant]/lim AND ('clinical trial'/de OR 'controlled clinical trial'/de OR 'feasibility study'/de OR 'intervention study'/de OR 'multicenter study'/de OR 'pilot study'/de OR 'randomized controlled trial'/de OR 'randomized controlled trial topic'/de)
