# Supplementary material for: Early Intervention Including an Active Motor Component in Preterms with Varying Risks for Neuromotor Delay: A Systematic Review and Narrative Synthesis
Source: J Clin Med. 2025 Feb 18;14(4):1364. doi: 10.3390/jcm14041364 (PMC11855987; doi:10.3390/jcm14041364)
Supplement: Supplementary file 1 [file jcm-14-01364-s001.zip › Supplementary material S2.pdf]

Supplementary material S2: theoretical frameworks and description of therapies

Description of theoretical frameworks

Different theoretical frameworks are used to design these early intervention programs with their own focus or aim such as family-centred practice, environmental enrichment, synactive theory of development or action-perception theory. Pure motor-based interventions are mainly focused on principles of motor learning and experienced-dependent neuroplasticity such as specificity, repetition, intensity and transfer. Additionally, it often includes the neural group theory , which highlights the importance of trail- and- error experiences to be able to create stable and flexible neural pathways for (motor) function. Family-centred interventions on the other side are also focusing on environmental enrichment implementing this to facilitate activity-dependent brain plasticity by providing additional social interaction, enhancing the parent-infant relationship and providing challenging activities. Similar, the action-perception theory focuses on the importance of the interaction with the environment to be able to understand the effects of your motor actions on the environment to improve problem-solving skills and the link between mind and body. Another popular theory is the synactive theory of development, in which early experience is highlighted as important factor for shaping development. Observations of the caregivers are of utmost importance to determine the needs of the infants and thus are highlighted in some interventions by using observation tools.

Descriptions of Therapies

1) Pure motor-based interventions

During the TETHERED KICKING [11] intervention, infants' lower limbs were connected to a mobile which rewarded the infant kicking movements by movements of the mobile.

EARLY CRAWLING [12], [13]interventions focus on self-initiated crawling movements of pre-crawling infants by positioning them in a prone position on a rolling crawling board (SIPPC or crawliskate). This crawling board encourages the movements of arms and legs by facilitating progression.

STICKY MITTENS [14] intervention consisted of one session of reaching stimulation by providing Velcro covered toys to the infant who was wearing open finger Velcro gloves. Spontaneous exploration of the toys was encouraged. Touching the toys, hence, resulted in sticking to the gloves and thus rewarding the infant for their effort.

ESPVM [15] (early stimulation program targeting visual and motor functions) is a homebased intervention program provided by the caregiver based on principles of activity-dependent learning and environmental enrichment. Visus was stimulated by

presenting visual contrast cards at static and moving positions. Motor function was stimulated by presenting toys and stimulating the infant to reach towards the object.

CARETOYSYSTEM[16] is a home-based tele-rehab system in which infants were placed in the play system and provided with motor, visual and auditory stimulations of different difficulties. The system itself registered the infant's behavior and progress and sent it to the rehab staff which made it possible for rehab staff to remotely monitor the training and adapt to training stimulations that provided the right level of challenge.

2) General family-centred interventions

COPCA [21, 25, 26, 27, 32, 35], (coping with and caring for infants with special needs) is a home-based intervention provided by the caregivers which are coached by a therapist. During this intervention, parents are encouraged to autonomously participate in the community and learned how to stimulate their infants functional mobility by challenging their motor behaviours based on the neural group theory. The caregivers were taught how to adapt the environment to provide trial-and-error experiences, and to accept atypical motor strategies in order to gain functional mobility.

SPEEDI [22, 24] (supportive play, exploration and early development intervention) is a two-phase intervention, starting at the NICU and continuing after discharge at home. During the first phase at NICU, parents are taught to identify ideal times to interact with their infants, and provide developmentally appropriate interaction. Additionally, they are asked to start to consider how the interaction will be with their infant after hospital discharge. In the second phase, a home-based intervention took place in which parents are supported by a physical or occupational therapist to provide daily opportunities to their child for motor and problem-solving based play. This was provided through environmental enrichment and active engagement based on principles of synactive theory of development and action-perception theory.

VIBesPlus program [29-31] a home-based intervention provided by a physiotherapist and a psychologist, aiming to promote infant development, to optimize parental mental health and to support families with important issues such as feeding or sleeping. The intervention mainly consists of education and information for parents about enriched environments, positive play interactions, infant behavioral cues, infant-parent relationship and developmental milestones. Additionally they learned techniques to improve motor development.

EARLY POST HOSPITAL PSYCHOMOTOR THERAPY [17] is a new physchomotor based therapy used in France based on the concept of intentionality which they defined as 'the fact of acting according to an intention, that is a representation of a future action not yet realized' also taking into account the cause-effects mechanisms of early developmental trajectories. The intervention consists of a clinical observation tool to

determine the care based on early signs of atypical development on different domains: sensorimotor integration, perceptual integration, relations, exploratory behaviour, tonic-emotional behaviour, self-regulation, motor organization and coordination. Both preventative as well as curative interventions are included to prevent or treat early signs of atypical development consisting of parental support to improve their parental sensitivity and self-confidence to identify the needs of their infant, as well as specific care for the infant focusing on positive bodily and emotional experiences, feelings an dynamic developmental processes.

EXPLORER BABY [18] is a holistic family-centred developmental support program for infants at-risk to improve exploratory play behaviours and motivation to move by environmental enrichment. Using observation and interaction between caregivers and experts, infants' temperament and interaction with the environment is taken into account and parents are learned how to adapt the environment to improve trail-and-error and exploratory behavior of the infant. Also taking into account body structure issues such as hypo- or hypertonia and its influence on play positions and exploratory play behaviour.

SAFE [19] (sensory strategies, activity-based motor training) is a family-centred intervention approach providing activity based motor training with sensory strategies and enriched environments carried out in the child's natural context. This program was based on the principles of neural group theory, dynamic system theory and principles of motor learning providing a large variety of activities adapted to their skills and encouraging parents to let the infant experience movements hands-off.

ENHANCED SOCIAL INTERACTION + ASSISTED EXERCISES PROGRAM [20]. Training sessions are provided to caregivers, instructing them on the implementation of social interaction activities such as singing, speaking, and storytelling, tailored to enhance caregiver-infant engagement. Additionally, caregivers learn to conduct guided infant exercises aligned with the NDT concept, while also gaining the ability to differentiate between appropriate and unsafe handling practices during these exercises.

Early intervention program of Ferreira et al. [23] provided support for early mother-childinteractions and opportunities for learning about child development conducted by a multiprofessional team. They used the Newborn behavioural Observation (NBO) tool to discover the infant's needs. Additionally, a baby book was provided with suggestions for developmental stimulation activities specific for each age range and different developmental domains. Finally, mothers groups were provided to share experiences about how to deal with challenges typical for the age of their child.

IBAIP [33] (Infant Behavioural Assessment and Intervention Program) uses the observation scale for infant behaviour to provide insights in the needs of the infant about self-regulatory competences, information exploration and improvement of development with a focus on environmental, behavioral and early developmental

factors. This intervention aims to support both infant and parent and is based on synactive theory of development and environmental enrichment.

The PREVENTIVE INTERVENTION PROGRAM [34] provides groups sessions based on IHDP to educate parents about infant-parent bonding, neurodevelopmental stimulation, modification of the environment, postural control and developmental milestones. Additionally, they provided home visits based on mother-infant transaction program to help parents implement their knowledge about understanding baby cues and to support in care.
