# Supplementary material for: Early Intervention Including an Active Motor Component in Preterms with Varying Risks for Neuromotor Delay: A Systematic Review and Narrative Synthesis
Source: J Clin Med. 2025 Feb 18;14(4):1364. doi: 10.3390/jcm14041364 (PMC11855987; doi:10.3390/jcm14041364)
Supplement: Supplementary file 1 [file jcm-14-01364-s001.zip › Supplementary material S3.pdf]

Table S1. overview of study results

(A). Study results of the motor-based interventions

**Author**	**Intervention groups**	**Between group**	**Within group**	**Subgroup analysis**
Campbell et al. 2015	Tethered kicking	No difference in overall average movement frequency<br>No differences in longitudinal changes	No differences in average movement frequency<br>TD: increase in movement frequency and proportion of synchronous kicks	CP and DD subjects lower amount of time and more difficulties to attain head in midline at 2mCA<br>Cp in control group: show declined frequency at 4m<br>TD greater proportion of synchronous movements at 4m compared to CP or DD<br>Proportion synchronous kicks increased over time (**TD>< CP** in control group)
	No intervention			
Dumuids-Vernet et al. 2023	Crawling training on the Crawliskate	**More traveled distance** (d=3.0)<br>**Better BSID-III at all assessment points**<br>**Better ASQ**<br>No differences for ATNAT (12m)<br>No differences in fine motor scores	**Increased BSID-III fine motor scaled scores over time**	
	Prone positioning on mattress	No differences in BSID-III with standard care		
	Standard care	No differences in BSID-III with mattress group		

Kolobe 2019	Reinforcement learning and error-based learning	**higher rotational amplitudes**<br>**higher wrist pathlengths** (vs TD)<br>No difference in footh pathlengths (vs reinforcement only)<br>**longer linear pathlength**	**Increase of rotational amplitude**<br>**increase of wrist path length**<br>no increase in foot path length<br>**increase of linear path length**<br>**increased scores on goal directed movements**
	Reinforcement learning only	No difference in wrist pathlengths	Similar rotational amplitude<br>No increase of wrist path length<br>No increase in foot path length<br>No increase in linear path length<br>Increased scores on goal directed movements
	Typical developing infants	**Lower wrist pathlengths** (vs error based learning)<br>larger increase in goal directed movements	**Increase of rotational amplitude**<br>**increase of wrist path length**<br>**increase of foot path length**<br>**increase in linear path length**<br>Increased scores on goal directed movements
Nascimento et al. 2019	Sticky mittens with open fingers	**Number of reaches:**<br>**more (post)**<br>no difference (retention)<br><br>Proximal adjustments:<br>**more bimanual reaches (**post and retention)<br>Distal adjustments: no differences<br>Grasping: no differences	Number of reaches:<br>increased (post)<br>decreased (retention)<br><br>Proximal adjustments:<br>increase in bimanual reaches<br>Distal adjustments: no differences<br>Grasping: no differences
	Spontaneous limb movements		Number of reaches:<br>decreased (retention)<br>Proximal adjustments:<br>similar over time<br>Distal adjustments: no differences

			Grasping: no differences	
Rodovanski et al. 2020	Standard care with additional information about early stimulation targeting visual and motor functions	No difference (Cramers V=0.25)	NI	
	Standard care			
Sgandurra et al. 2017	CareToy System	**IMP stronger improvements (d=0.69)**<br>AIMS: trends towards better scores (d=0.33)<br>No differences at follow-up (both received CareToy with different start timing)	NI	
	Standard care			
**(B). Study characteristics of the family-centred interventions**				
**Author**	**Intervention groups**	**Between group**	**Within group**	**Subgroup analysis**
Alberge et al. 2023	Psychomotor therapy	**better motor composite score** at 9m<br>No differences in BSID-III scores at 24m<br>No differences in neurological outcome at 24m	NI	Maternal education: **benefit of intervention** for low maternal education group
	Standard care	More additional physiotherapy support received<br>Beter fine motor skills		
Altunalan et al. 2023	Explorer Baby	No differences	Beneficial effects across all developmental subskills<br>No differences in percentage of developmental delay	
	NDT		Beneficial effects across all developmental subskills<br>No differences in percentage of developmental delay	

Apaydin et al. 2023	SAFE early intervention	**stronger improvements in TSFI and AHMED-IS**<br>scores **better TSFI and AHEMD-IS** at T2<br>no interaction effect for BSID-motor, COMP	**improvements on TSFI, AHEMD-IS, BSID-motor and COMP**
	NDT		No improvements in TSFI<br>**improvements for AHEMd-IS and COMP**
Cooper et al. 2020	Assisted-exercise (based on NDT) and enhanced social interaction program	NI	**Reduction in TIMP z-score** decrease between baseline and 3m
	Enhanced social interaction alone		
Dirks et al. 2016	COPCA	**more often sitting position** during bathing (6m)<br>no sign differences in bathing position (18m)	NI
	Traditional infant physiotherapy (mainly based on NDT)	no sign differences in bathing position (18m)	
Dusing et al. 2018	SPEEDI + Standard care	Duration of toy contact:<br>No group x time interaction<br>No group difference<br>ES suggest small but measurable effect of the intervention (ES_{post}: 0.11, ES_{FU1}= 0.41 and ESFU2= 0.38)	**Increased duration of toy contact**<br>Neuromotor control and development improved with large ES (d=1.04) for TIMPS and BSID-motor (d=1.2) (9m and 12m)
	Standard care		**Increased duration of toy contact**<br>Neuromotor control and development improved with large ES (d=1.04) for TIMPS and BSID-motor (d=1.2) (9m and 12m)
Ferreira et al. 2020	Standard care + early intervention	**lower gross motor mean scores** at 12m<br>Differences decreases over time	Fine motor differences-in-mean-scores increase

		Lowest prevalence of developmental delay in fine motor skills at 12m<br>No differences in prevalence of developmental delay	Gross motor differences-in-mean-scores decrease
	Standard care	**Higher gross motor mean scores**<br>**Higher fine motor mean scores**	
Finlayson et al. 2020	SPEEDI + standard care	**Less infants with absent fidgety GM (**at 3m)<br>No difference in TIMP score (at 3m) (d=0.09)<br>**better gross motor score** on BSID (at 4m) (d=0.57)<br>minimal difference for fine motor score (d=0.83)	Improvement of TIMP scores over time
	Standard care		
Hamer et al. 2017	COPCA	No differences	NI
	Traditional infant physiotherapy (mainly based on NDT)		
Hielkema et al. 2020	COPCA	No interaction effects of intervention with age<br>differences	NI
	Traditional infant physiotherapy		
Kara et al. 2019	Family-based intervention based on COPCA	No between group interaction in fine and gross motor development<br>No differences between groups in improvements over time	**improvement of fine motor skills**<br>**improvement of gross motor skills**
	Traditional early intervention		**improvement of fine motor skills**<br>**improvement of gross motor skills**
Ochandorena-Acha et al. 2022	Early physiotherapy intervention program + standard care	No differences	No differences

	Standard care (NIDCAP)			
Spittle et al. 2016	Preventative care program (VIBeS program) + standard care<br>Standard care	No differences (between preterm groups)<br>**VPT group performing below TD peers** (at 8 years)<br>No differences (preterm)	NI	
Spittle et al. 2018	Preventative care program (VIBeS program) + standard care<br>Standard care	No differences on BSID-III (at 2y) or mABC-2 (at 4 and 8 y) for the total group	NI	Different intervention effects according to social risk:<br>Higher social risk group: more effect of intervention on cognitive function, language, motor function, behavioral problems
Stedall et al. 2022	Preventative care program (VIBeS program) + standard care<br>Standard care	No differences in prevalence of CP<br>Little evidence of benefits for executive functioning<br>No differences in motor scores	NI	High social risk group: stronger effect of intervention on total motor and balance
Van Balen et al. 2019	COPCA	No differences in postural adjustments	Direction specificity: **number of direction specific postural activity increased**<br><br>Muscle recruitment strategies: **bottom up recruitment decreased**<br>No increase in top-down recruitment<br><br>Anticipatory activation: less anticipatory trunk muscle activation 18m vs 4m; no overall decrease<br><br>latency: **Slower latency** at 6m vs 4m	

	Traditional infant physiotherapy (mainly based on NDT)		Direction specificity:<br>No increase of direction specificity at trunk or neck level<br><br>Muscle recruitment:<br>Bottom up recrtuitment decreased<br>Increase in top-down recruitment<br><br>Anticipatory activation:<br>Overall lower anticipatory activation rate at 18m (vs 6m)<br><br>Latency:<br>Slower latency at 18m (vs 4m)	
Van Hus et al. 2016	Infant Behavioral Assessment and Intervention Program (IBAIP)	Differences in z scores between group did not change over time	Intervention effect	BPD: **larger intervention effect (ES= 0.9)**<br>Low maternal education: **small intervention effect (ES= 0.4)**
	Standard care		Intervention effect	
Youn et al. 2021	Preventive intervention program	No differences	NI	
	Standard care			
Ziegler et al. 2021	COPCA	IMP: more improvements at variation and performance (18m); no differences (3&6m)<br>**time x group interaction: improved 12 % points more** between 3m and 18m<br>BSID-III: no differences at 2y<br>PEDI : no differences (at 3 and 18m)	**IMP improvement in the mean variation domain** between 3 and 6m<br>**Improvement in Performance domain**: between 3 and 6m after baseline and 18m	
	Standard care	IMP performance and variation: no differences at 3&6m<br>PEDI : higher functional mobility score (at 6m)	**IMP improvement in the mean variation domain** between 3 and 6m	

	**Improvement in Performance domain**: between 3 and 6m after baseline and 18m

Abbreviations: TD: typical developing infant; CP: cerebral palsy; DD infants with delayed development: NDT, neurodevelopmental treatment; SAFE, Sensory strategies, Activity-based motor training, Family collaboration, and Environmental Enrichment; COPCA, COPing and CAring for infants with special needs; SPEEDI, Supporting Play Exploration and Early Developmental Intervention; VIBeS, Victorian Infant Brain Studies; IBAIP, Infant Behavioral Assessment and Intervention Program; BPD: bronchopulmonary dysplasia; N, number; NA, not applicable; NI, no information; CA, corrected age, m: months; TIMP, Test of Infant Motor Performance; AIMS, Alberta Infant Motor Scale; GMA, general movements assessment; ATNAT, Amiel-Tison Neurological Assessment; ASQ, Ages and Stages Questionnaire; IMP, Infant Motor Profile; COMP, Canadian Occupational Performance Measure; PEDI, Pediatric Evaluation of Disability Inventory; VABS, Vineland Adaptive Behavior Scales; DCD-Q, Developmental Coordination Disorder Questionnaire; GMFM, Gross Motor Function Measure; M-ABC-2, Movement Assessment Battery for Children, 2nd version;
